# Differential susceptibility of SARS‐CoV‐2 in animals: Evidence of ACE2 host receptor distribution in companion animals, livestock and wildlife by immunohistochemical characterisation

**DOI:** 10.1111/tbed.14232

**Published:** 2021-07-26

**Authors:** Fabian Z. X. Lean, Alejandro Núñez, Simon Spiro, Simon L. Priestnall, Sandra Vreman, Dalan Bailey, Joe James, Ethan Wrigglesworth, Alejandro Suarez‐Bonnet, Carina Conceicao, Nazia Thakur, Alexander M.P. Byrne, Stuart Ackroyd, Richard J. Delahay, Wim H. M. van der Poel, Ian H. Brown, Anthony R. Fooks, Sharon M. Brookes

**Affiliations:** ^1^ Department of Pathology and Animal Sciences Animal and Plant Health Agency (APHA) Addlestone Surrey UK; ^2^ Wildlife Health Services Zoological Society of London London UK; ^3^ Department of Pathobiology and Population Sciences The Royal Veterinary College North Mymms UK; ^4^ Wageningen Bioveterinary Research Lelystad The Netherlands; ^5^ The Pirbright Institute Woking Surrey UK; ^6^ Department of Virology APHA Addlestone Surrey UK; ^7^ National Wildlife Management Centre APHA, Sand Hutton York UK

**Keywords:** ACE2, felids, immunohistochemistry, mustelids, SARS‐CoV‐2

## Abstract

Angiotensin converting enzyme 2 (ACE2) is a host cell membrane protein (receptor) that mediates the binding of coronavirus, most notably SARS coronaviruses in the respiratory and gastrointestinal tracts. Although SARS‐CoV‐2 infection is mainly confined to humans, there have been numerous incidents of spillback (reverse zoonoses) to domestic and captive animals. An absence of information on the spatial distribution of ACE2 in animal tissues limits our understanding of host species susceptibility. Here, we describe the distribution of ACE2 using immunohistochemistry (IHC) on histological sections derived from carnivores, ungulates, primates and chiroptera. Comparison of mink (*Neovison vison*) and ferret (*Mustela putorius furo*) respiratory tracts showed substantial differences, demonstrating that ACE2 is present in the lower respiratory tract of mink but not ferrets. The presence of ACE2 in the respiratory tract in some species was much more restricted as indicated by limited immunolabelling in the nasal turbinate, trachea and lungs of cats (*Felis catus*) and only the nasal turbinate in the golden Syrian hamster (*Mesocricetus auratus*). In the lungs of other species, ACE2 could be detected on the bronchiolar epithelium of the sheep (*Ovis aries*), cattle (*Bos taurus*), European badger (*Meles meles*), cheetah (*Acinonyx jubatus*), tiger and lion (*Panthera spp*.). In addition, ACE2 was present in the nasal mucosa epithelium of the serotine bat (*Eptesicus serotinus*) but not in pig (*Sus scrofa domestica*), cattle or sheep. In the intestine, ACE2 immunolabelling was seen on the microvillus of enterocytes (surface of intestine) across various taxa. These results provide anatomical evidence of ACE2 expression in a number of species which will enable further understanding of host susceptibility and tissue tropism of ACE2 receptor‐mediated viral infection.

## 1 INTRODUCTION

Angiotensin I converting enzyme 2 (ACE2) is a component of the renin‐angiotensin‐aldosterone system which plays a critical role in the homeostasis of blood pressure through its regulation of hydrolysis of angiotensin II (Hamming et al., 2007). ACE2 is typically expressed in the lung and kidney as demonstrated by a combination of transcriptomic and immunohistochemical (IHC) studies of human tissues (Hikmet et al., 2020; Ortiz et al., 2020). The spatial distribution of ACE2 expression in animals is less well understood, being limited to experimental laboratory animals including mustelids and non‐human primates (Gembardt et al., 2005; Lee et al., 2020a; van den Brand et al., 2008; Zheng et al., 2020).

The susceptibility of companion animals to severe acute respiratory syndrome (SARS) coronavirus infection was first recognised in domestic cats (*Felis catus*) living among infected humans during the 2003 SARS‐CoV outbreak in Hong Kong (Martina et al., 2003). The global spread of SARS‐CoV‐2 in the human population has been accompanied by naturally acquired infections in pet cats and dogs (*Canis lupus familiaris*) (Barrs et al., 2020; Segales et al., 2020; Sit et al., 2020), captive tigers (*Panthera tigris*), lions (*Panthera leo*), snow leopard (*Panthera uncia*) and puma (*Puma concolor*) from zoological collections (McAloose et al., 2020; O.I.E, 2021; USDA, 2020), ferrets (*Mustela putorius furo*) (Gortázar et al., 2021) and in farmed and wild American mink (*Neovison vison*) (Molenaar et al., 2020; O.I.E, 2021). In most cases, animals developed self‐limiting, low grade respiratory diseases (sub‐clinical) which did not require further veterinary intervention. However, widespread infection in intensively housed mink has been associated with clinical disease and mortality (Oreshkova et al., 2020). Critically, outbreaks of SARS‐CoV‐2 infection in mink farms have resulted in the emergence of novel virus variants with potential consequences for public health (Hoffmann et al., 2021; Larsen et al., 2021; Oreshkova et al., 2020; Oude Munnink et al., 2021), which has also been reported in experimentally infected ferrets (Everett et al., 2021; Richard et al., 2020).

The variability of host susceptibility to SARS‐CoV‐2 is still not completely understood. At the cellular level, the spike glycoprotein of the virus has demonstrated a broad host cellular receptor tropism with strong affinity for dogs, cats, wild felids, sheep and cattle ACE2 based on surrogate viral entry assays and live virus infection studies (Conceicao et al., 2020; Liu et al., 2021). In addition, *in vivo* studies have provided evidence of a productive infection (live virus) in cats in the absence of disease, but experimental infection could not be successfully established in dogs and cattle (*Bos taurus*) (Bosco‐Lauth et al., 2020; Ulrich et al., 2020). Although predominantly presented as a respiratory disease, SARS‐CoV‐2 can regularly be detected in rectal swabs of naturally infected dogs or experimentally infected ferrets (Everett et al., 2021; Sit et al., 2020) and infection of bat intestinal organoids can result in productive virus replication (live virus) (Zhou et al., 2020). This observation also extends to other coronaviruses that are capable of infecting both the respiratory and gastrointestinal tract of animals (Saif & Jung, 2020).

Currently there is a gap in our knowledge regarding the anatomical distribution of ACE2 proteins in a broader range of animal hosts and, thus, data will be required in order to further our understanding of host range susceptibility (Delahay et al., 2021). The current study reports on the detection of ACE2 by IHC in several mammalian species including companion animals, livestock and wildlife, with a focus on the lung and small intestine. In addition, the differential distribution of ACE2 in the entire respiratory tract (upper and lower) of mink, ferrets, dogs, cats and golden Syrian hamster (*Mesocricetus auratus*) are also described to investigate the relationships between the cognate virus receptor and host susceptibility.

## 2 MATERIALS AND METHODS

### 2.1 Transfection of cells

The production of species‐specific ACE2 in cells was conducted as previously described (Conceicao et al., 2020). Briefly, codon optimised ACE2‐expressing plasmids from different animals were synthesised and cloned into pDISPLAY (BioBasic). A total of 500 ng of a subset of ACE2 expression constructs (human, hamster, ferret, dog, cat, pig, myotis and rhinolophus bats) or an empty vector for mock transfection (pDISPLAY) in OptiMEM (Gibco) were introduced into 24‐well plated baby hamster kidney (BHK‐21) cells using TransIT‐X2 transfection reagent (Mirus). These cells have previously been confirmed for transfection by western blot, flow cytometry and SARS‐CoV‐2 pseudo‐type virus infections (Conceicao et al., 2020). Monolayers of *in vitro* transfected cells were fixed in 10% neutral‐buffered formalin for 24 h, re‐suspended and centrifuged at 1500 × g. Following removal of the supernatant, cell pellets were re‐suspended in 2% agarose (Sigma) and allowed to set. Agarose‐embedded cell pellets were then processed into formalin‐fixed paraffin embedded sections by routine histology methods (Lean et al., 2020).

### 2.2 Sequence analysis

ACE2 amino acid sequences were obtained from GenBank and the immunogen sequence of a commercial anti‐ACE2 antibody (Abcam ab15348) was derived from the manufacturer's datasheet. Amino acid sequence alignment was conducted using Protein BLAST available on blast.ncbi.nlm.nih.gov and visualised using MEGA7 (Kumar et al., 2016).

### 2.3 Immunohistochemistry

Formalin‐fixed tissues were obtained from histology archives held by the Animal and Plant Health Agency, the Zoological Society of London and the Royal Veterinary College, UK and Wageningen Bioveterinary Research, Netherlands. These tissues were collected during necropsy as part of veterinary investigations or from experimental control animals that were not challenged with pathogens. The only exception was that a subset of ferrets infected with SARS‐CoV‐2 (Everett et al., 2021) was included in this study to facilitate comparison of ACE2 expression between infected and non‐infected hosts. Animals with known disease or tissues with poor preservation state were excluded from this study. Anti‐ACE2 antibody was validated for IHC using BHK‐21 cells expressing species‐specific ACE2. Sections of 4 μm thickness were dewaxed and rehydrated through xylene and absolute alcohol, respectively, quenched for endogenous peroxidase with 3% hydrogen peroxide in methanol (VWR International) for 15 min at room temperature (RT), before epitope unmasking using a pH 6 buffer (citric acid monohydrate; Fisher Scientific, adjusted to pH 6 with 1 M sodium hydroxide; VWR International) for 18 min at 100°C by microwave. Slides were blocked with normal goat serum for 30 min at RT (1/66 dilution; Vector Laboratories) and assembled into cover plates to facilitate IHC using the Shandon Sequenza system (Shandon). Samples were then incubated with a rabbit polyclonal ACE2 (Abcam ab15348) primary antibody (van den Brand et al., 2008) or with a matching isotype control (rabbit IgG Vector Laboratories, UK) at 1 μg/mL, on serial tissue section, for 1 h at RT. This was followed by incubation with rabbit‐specific Envision+™ HRP‐labelled polymer (Dako) with an additional normal goat serum (1/66 dilution; Vector Laboratories) for 30 min at RT and visualised using 3,3‐diaminobenzidine tetrahydrochloride (Sigma Aldrich) for 10 min at RT. Tris‐buffered saline (0.85% NaCl) with Tween (Fisher Scientific, VWR International) was used for rinsing sections between incubations and as the antibody diluent. Subsequently, sections were counterstained within Mayer's haematoxylin (Surgipath), dehydrated and cleared in absolute alcohol and xylene, and glass coverslips mounted using dibutyl phthalate xylene (TCS Biosciences).

## 3 RESULTS

### 3.1 Assessment of antibody cross‐reactivity with ACE2 of diverse mammalian species

**FIGURE 1:**
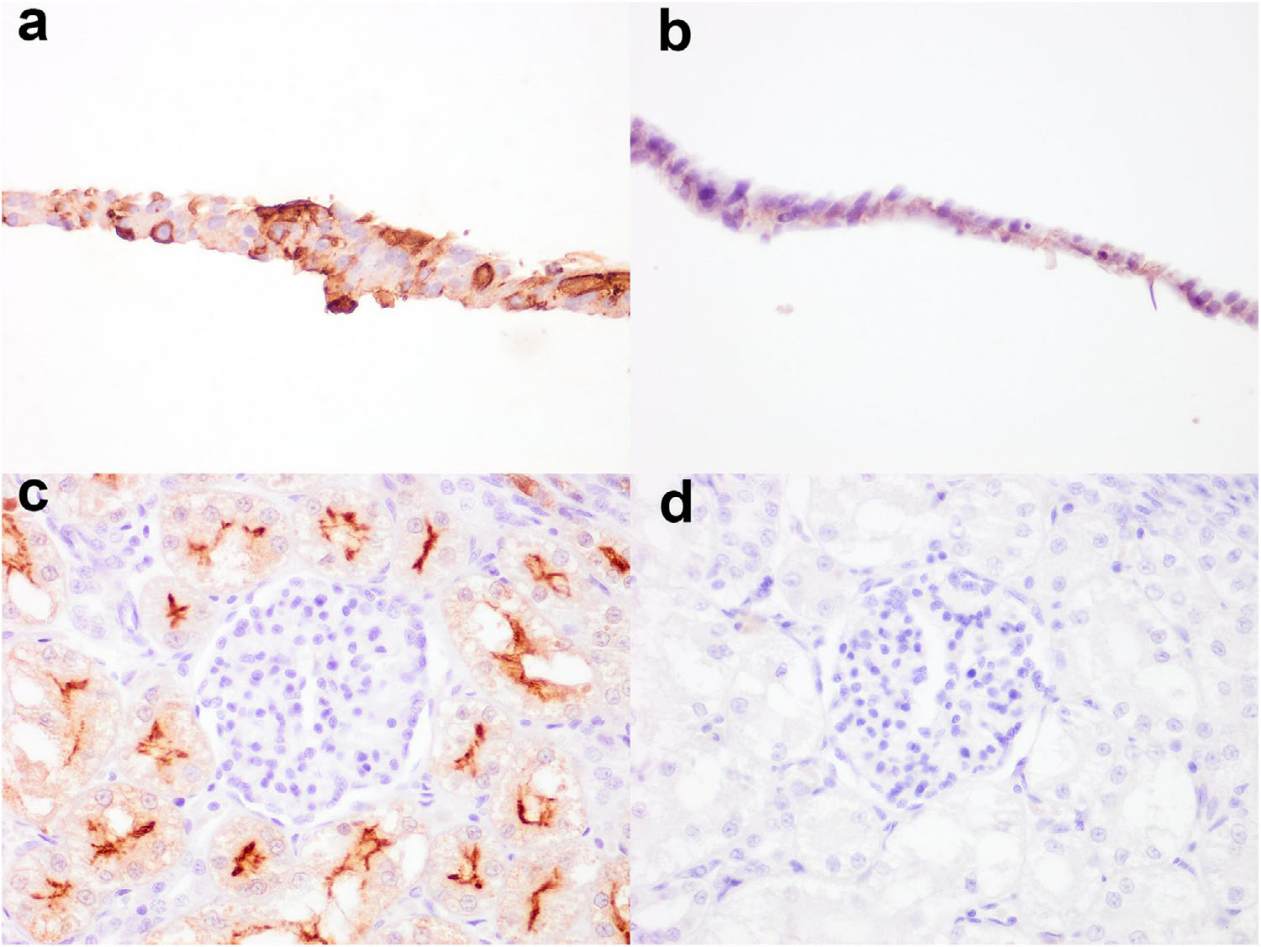
Confirmation of ACE2 immunolabelling specificity using ACE2 transfected BHK‐21 cells and kidney sections. Immunolabelling performed with anti‐ACE2 rabbit polyclonal antibody on BHK‐21 cells transfected with ferret (*Mustela putorius furo*) ACE2 plasmid (a) or no plasmid control (b). To evaluate for false immunoreactivity, serial sections of kidney, in this case from Sumatran tiger (*Panthera tigris sondaica*), were immunolabelled with anti‐ACE2 primary antibody or concentration matched rabbit IgG. Positive membranous labelling can be detected in the apical aspect of the renal tubular epithelium when probed with anti‐ACE2 primary antibody (a) but not with rabbit IgG (d). Images taken at 400× magnification

ACE2 IHC was optimised in accordance with a previous published protocol (van den Brand et al., 2008). Subsequently, the IHC was evaluated for cross‐reactivity with ACE2 of other species, first using BHK‐21 cells expressing species‐specific plasmid‐driven ACE2 proteins. Intense membranous and variable cytoplasmic immunolabelling was present on cells expressing ACE2 protein of ferret (Figure 1a), dog, cat, golden Syrian hamster, pig (*Sus scrofa domestica*), little brown bat (*Myotis lucifugus*) and least horseshoe bat (*Rhinolophus pusillus*) (Supporting information Table S1). There was no labelling present on empty plasmid control cells (Figure 1b). In addition to validation of the IHC technique with ACE2 expressing cells, kidney sections from each included species were used as positive controls to verify immunolabelling. This was characterised by intense membranous immunolabelling on the apical aspect of the renal tubular epithelium (Figure 1c). The species evaluated included dog, cat, pig, cattle, sheep (*Ovis aries*), horse (*Equus caballus*), cheetah (*Acinonyx jubatus*), Sumatran (*P. tigris sondaica*; Figure 1c) and Siberian tiger (*P. tigris altaica)*, Asiatic and African lion (*P. leo leo* and *P. leo*, respectively), European lynx (*Lynx lynx*), Western gorilla (*Gorilla sp*.), American mink, ferret, European badger (*Meles meles*), golden headed lion tamarin (*Leontopithecus chrysomelas*) and common marmoset (*Callithrix jaccus*) (Supporting information Table S1). Non‐specific immunolabelling was not observed when anti‐ACE2 primary antibody was replaced with a concentration matched rabbit IgG for each species described above (Figure 1d).

ACE2 amino acid homology analysis of the species of interest was aligned to the immunogen sequence of human ACE2 used for the generation of the polyclonal antibody that was applied for IHC. The amino acid sequences were highly conserved among all the species assessed in the present study. This included 100% homology with the Western gorilla, 94% for horse, ferret and American mink, 89% for little brown bat, least horseshoe bat, domestic and wild felids (tiger and cheetah), dog and sheep, 83% for cattle, 78% for pig and common marmoset, and 67% for hamster (Supporting informationTable S2). The immunogen sequence was located at the C‐terminus of the ACE2 protein within the cytosolic domain. Whilst there was high conservation in the immunogen sequences across the selected species, there were a number of amino acid substitutions, in particular at position 798 (threonine in human ACE2) (Supporting information Table S2). However, whilst at a biochemical level some of these substitutions may be individually unfavourable (Russell et al., 2003), they do not appear to have an overall effect on anti‐ACE2 antibody binding, as supported by IHC validation on BHK‐21 cells expressing species‐specific ACE2 (Figure 1; Supporting information Table S1).

### 3.2 Differential distribution of ACE2 in the respiratory tract of the mink, ferret, dog, cat and hamster

**FIGURE 2:**
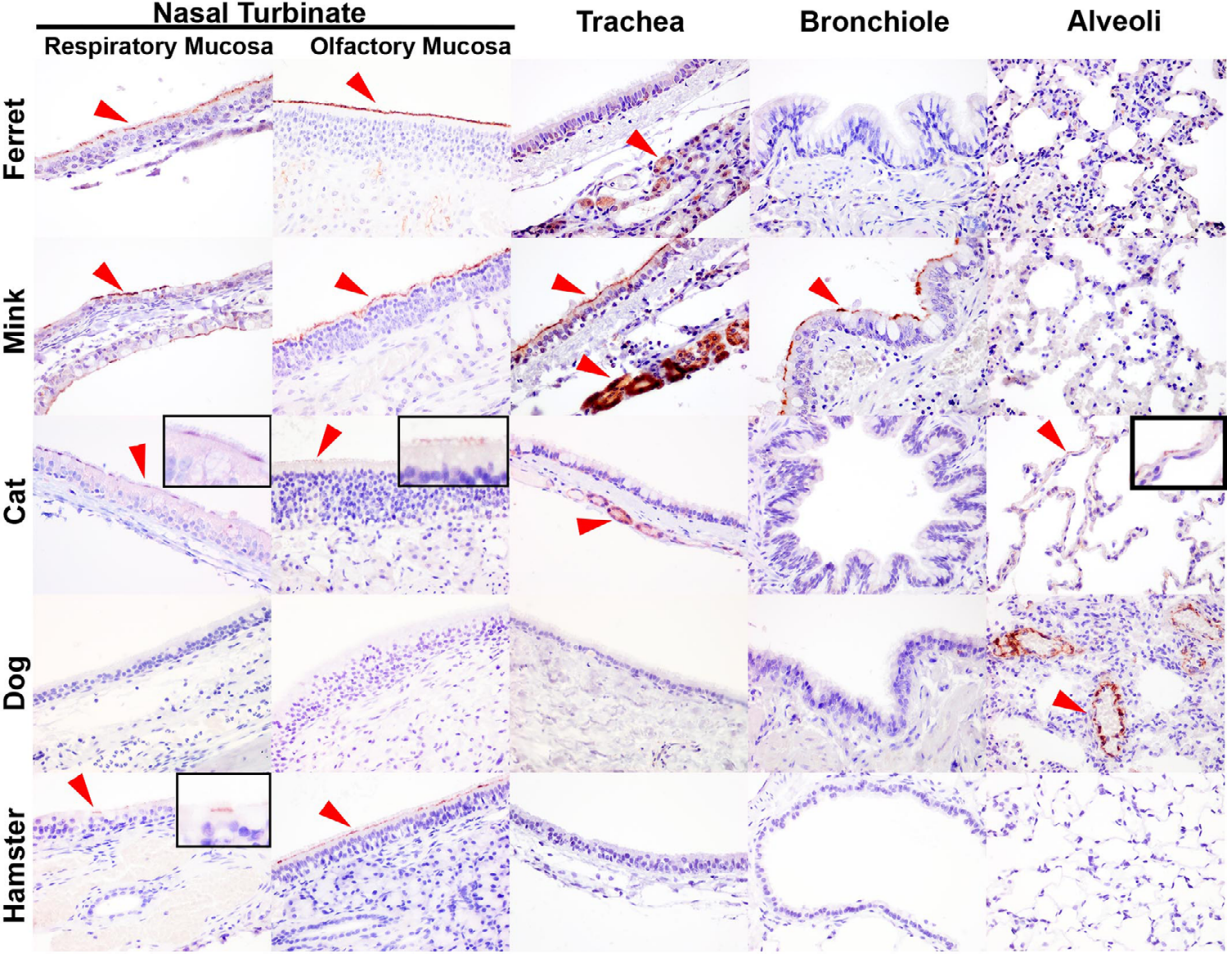
Immunohistochemical comparison of ACE2 distribution in the upper and lower respiratory tract of the ferret (*Mustela putorius furo*), mink (*Neovison vison*), cat (*Felis catus*), dog (*Canis lupus familiaris*) and golden Syrian hamster (*Mesocricetus auratus*). Positive immunolabelling indicated with red arrow heads. In the ferret, ACE2 was only detected in the cilia of the epithelium within the nasal turbinate (respiratory and olfactory mucosa) and the tracheal submucosal glands. In the mink, ACE2 was found on the cilia of the respiratory epithelium in the nasal turbinate (respiratory and olfactory mucosa), trachea and bronchiole, and also in the tracheal submucosal glands. In the cat, ACE2 immunolabelling was weak and represented by a discontinuous labelling pattern on the respiratory and olfactory mucosal cilia, and infrequent detection in the tracheal submucosal glands and type I pneumocytes. In the dog, ACE2 was absent in the airway passages and was only detectable in endothelium and tunica media of the medium‐sized vessels within the lung. In the hamster, ACE2 was infrequently detected in the respiratory and olfactory mucosa within the nasal turbinate. Images taken at 400× magnification. Insets referred to sparsely positive areas

Experimental infection of ferrets with SARS‐CoV‐2 resulted in infection in the upper respiratory tract and virus shedding, as confirmed by IHC, in situ hybridisation, virus isolation and polymerase chain reaction (PCR), but with no evidence of virus‐mediated pneumonia (Everett et al., 2021; Schlottau et al., 2020; Shi et al., 2020). In contrast, widespread infection has been observed in farmed minks with associated viral infection of the lung and sequelae of pneumonia (Molenaar et al., 2020). In the present study, we evaluated upper and lower respiratory tract sections from the mink and ferrets (Figure 2). IHC analysis of tissues from experimentally infected ferrets and from a healthy uninfected ferret revealed the presence of abundant ACE2 in the respiratory and olfactory mucosa epithelium of the nasal turbinate, but absence in the lower respiratory tract (encompassing the trachea, bronchus, bronchioles and alveoli). In contrast, immunolabelling was detected throughout the whole respiratory system of the mink, including the nasal turbinate (both respiratory and olfactory mucosa), trachea, bronchus and bronchiole but not the alveoli. The cell population positive for ACE2 in the mink included the columnar respiratory epithelial cells within the air passages and also the tracheal submucosal glands.

SARS‐CoV‐2 infection in the dog and cat have been reported in both household and experimental settings but without clinical disease (Barrs et al., 2020; Gaudreault et al., 2020; Segales et al., 2020; Shi et al., 2020; Sit et al., 2020). Based on IHC, ACE2 protein expression was only occasionally detected in the nasal respiratory and olfactory mucosa epithelium in the nasal turbinate of the cat, in which the labelling pattern was discontinuous across the cilia (Figure 2), in contrast to the uniform labelling in the nasal mucosa of the ferret and mink. Weak immunolabelling was also detected in the tracheal submucosal glands of the cat and in rare type I pneumocytes. In the dog, ACE2 immunolabelling was only detected in the endothelium and tunica media of medium‐sized vessels in the lung and absent in the respiratory tract. ACE2 distribution in the golden Syrian hamster was also evaluated due to the high susceptibility to SARS‐CoV‐2 infection and the frequent utility as an experimental model (Chan et al., 2020). In this species, ACE2 was only infrequently detected in the epithelium of the nasal mucosa. It was also noted that immunolabelling was marginally more abundant in the epithelium of the olfactory mucosa than in the nasal respiratory mucosa (Figure 2). However, ACE2 was absent in the trachea, bronchiole and alveoli of the hamster.

Overall, the findings highlighted the upper respiratory tract, specifically the nasal turbinates, as a potentially highly susceptible tissue for SARS‐CoV‐2 infection in the ferret, mink, cat and hamster. The presence of ACE2 in the lower respiratory tract of the mink and cat can also be related to the risk of developing disease following SARS‐CoV‐2 infection.

### 3.3 Detection of ACE2 in the lung and small intestine of other animals and the nasal mucosa of a subset of species

**FIGURE 3:**
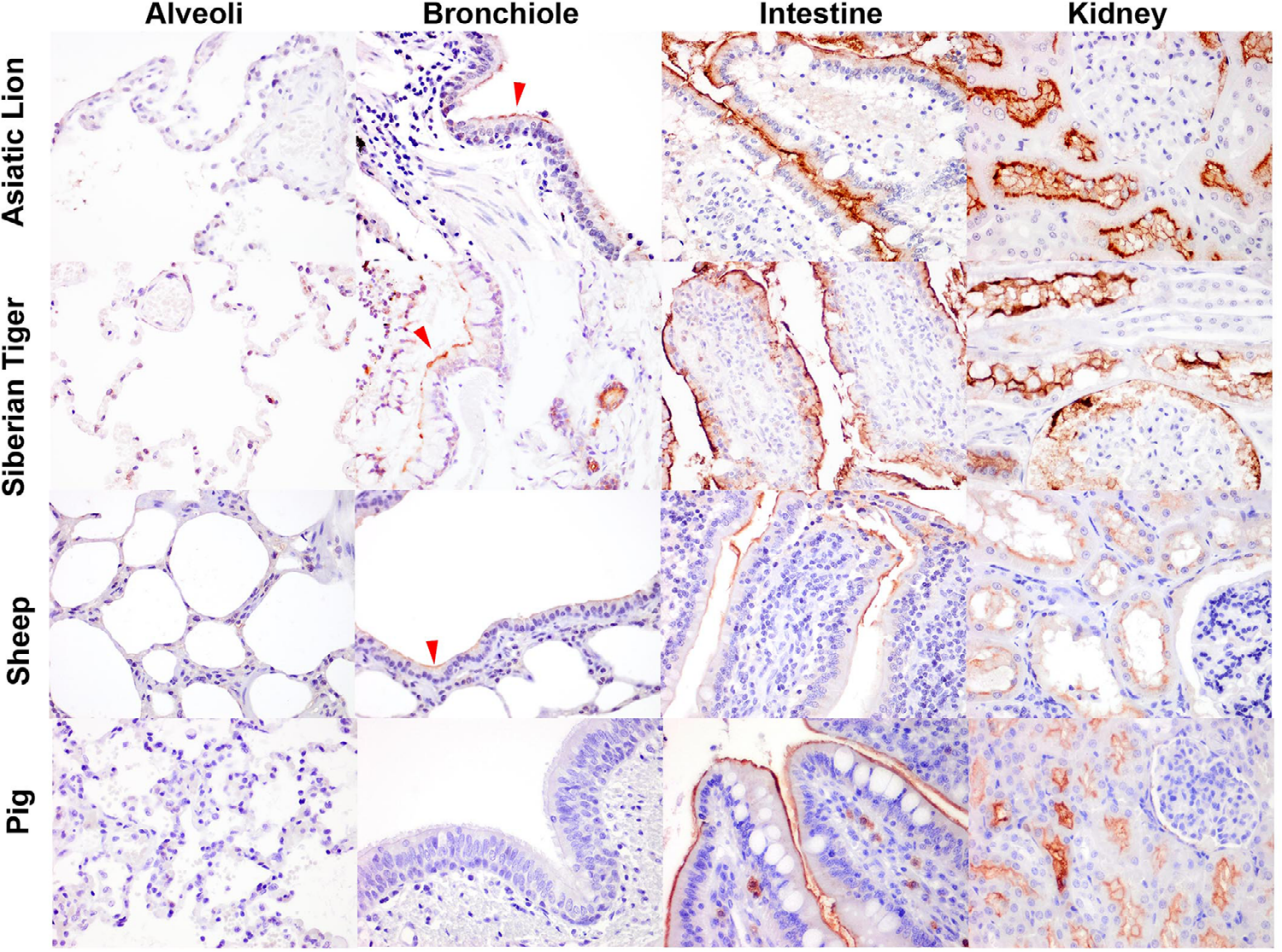
Immunohistochemical characterisation of ACE2 distribution in the lung, small intestine and kidney of large felids and livestock. Positive immunolabelling of ACE2 was observed in the cilia of the bronchiolar epithelium (red arrow heads) in the Asiatic lion (*Panthera leo leo*), Siberian tiger (*Panthera tigris altaica*) and sheep (*Ovis aries*). In addition, ACE2 was detected on the brush border of the intestinal and renal tubular epithelial cells. Images taken at 400× magnification

**FIGURE 4:**
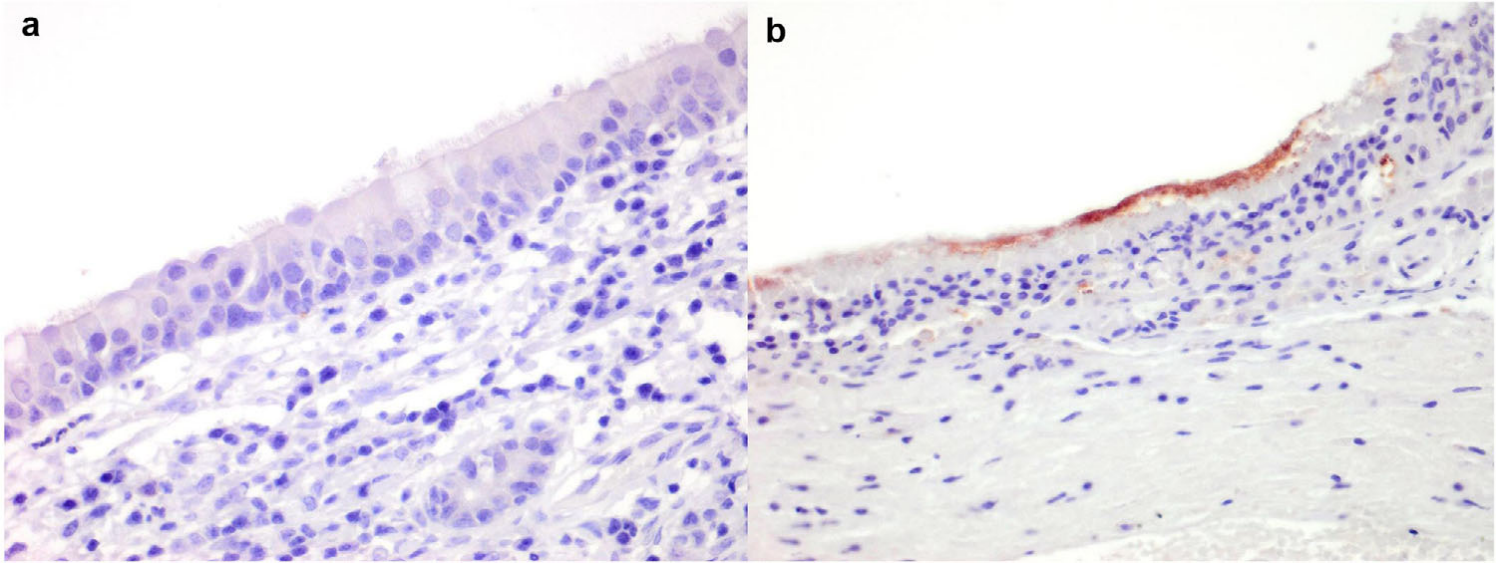
Immunohistochemical characterisation of ACE2 distribution in the nasal turbinate. Absence of immunolabelling in the pig (a), positive immunolabelling in the serotine bat (*Eptesicus serotinus*) (b)

Histological sections of the lungs and intestine of diverse mammalian species were evaluated for the presence of ACE2 by IHC (Table 1). For most species, the lung was the only respiratory tissue examined because this is one of the most common samples collected at necropsy and as pneumonia is implicated in human cases of COVID‐19 and laboratory animal models of SARS‐CoV‐2 infection (Bussani et al., 2020; Chan et al., 2020). In addition, the small intestine was examined because of the potential of enteric infection and shedding of virus in faeces (Lamers et al., 2020; McAloose et al., 2020; Molenaar et al., 2020). In the alveoli, ACE2 was only detected in the pneumocytes of cats, and not identified in the alveolar cells of any other species (Figure 2). However, the endothelium and tunica media of the medium‐sized blood vessels within the alveolar septa of the dog, Sumatran tiger and cheetah (Figures 2 and 3, Supporting information Figure S1) were immunopositive for ACE2. In contrast, ACE2 was detected on the apical aspect or cilia of the bronchiolar epithelium of domestic cattle and sheep, mink, European badger, Sumatran and Siberian tiger, African and Asiatic lion; and also bronchioles of the mink. No ACE2 immunolabelling was detected in the bronchioles of the pig, horse, ferret, Western gorilla, golden headed lion tamarin and the common marmoset (Supporting information Figures S1 and S2). In the intestines, ACE2 was detected in all species investigated, whereby the immunolabelling was ubiquitous on the brush border of the epithelial cells on the tip of the intestinal villi, and was infrequent to non‐existent in the crypts of the villi (Figure 3, Table 1, Supporting information Figures S1 and S2). Histological sections of the nasal mucosa of a limited number of mammalian species were evaluated for ACE2 by IHC, owing to the infrequent sampling of the upper respiratory tract. In addition to the mink, ferret, dog, cat and hamster described above, ACE2 expression was absent in the nasal mucosa of the pig (Figure 4a), cattle and sheep (data not shown), but present on the mucosa of the serotine bat (*Eptesicus serotinus*) (Figure 4b).

**TABLE 1:** Immunohistochemical characterisation of ACE2 in the lung and small intestine of companion animals, livestock and wild mammals

Common name *Scientific name* (Sample size)	Alveoli	Bronchiole	Small intestine*	*In vivo* evidence of host susceptibility
Cattle *Bos Taurus* (n = 1)	–	+, bronchiole epithelium	n/a	Experimental, asymptomatic (Falkenberg et al., 2021; Ulrich et al., 2020)
Sheep *Ovis aries* (n = 1)	–	+, bronchiole epithelium	+	–
Alpaca *Vicugna pacos* (n = 1)	–	–	n/a	–
Pig *Sus scrofa domestica* (n = 2)	–	–	+	Experimental, asymptomatic (Schlottau et al., 2020; Vergara‐Alert et al., 2020)
Dog *Canis lupus familiaris* (n = 2)	+, endothelium	–	+	Field and experimental, asymptomatic (Bosco‐Lauth et al., 2020; Sit et al., 2020)
Cat *Felis catus* (n = 5)	+, type I pneumocyte	–	+	Field and experimental, asymptomatic and symptomatic (Barrs et al., 2020; Bosco‐Lauth et al., 2020)
Horse *Equus caballus* (n = 1)	–	–	+	–
Ferret *Mustela putorious furo* (n = 3)	–	–	+	Field and experimental, asymptomatic (Carvallo et al., 2021; Everett et al., 2021; Gortázar et al., 2021; Schlottau et al., 2020; Shi et al., 2020)
American mink *Neovison vison* (n = 2)	–	+, bronchiole epithelium	+	Field and experimental, pneumonia (Molenaar et al., 2020; Shuai et al., 2020 )
Golden Syrian hamster *Mesocricetus auratus* (n = 3)	–	–	+	Experimental, pneumonia (Chan et al., 2020; Gerhards et al., 2021)
Cheetah *Acinonyx jubatus* (n = 1)	+, endothelium	–	n/a	–
Sumatran tiger *Panthera tigris sondaica* (n = 1)	+, endothelium	+, bronchiole epithelium	+	Field, cough and wheezing (McAloose et al., 2020)
Siberian tiger *Panthera tigris altaica* (n = 2)	–	+, bronchiole epithelium	+	Field, cough and wheezing (McAloose et al., 2020)
Asiatic lion *Panthera leo leo* (n = 1)	–	+, bronchiole epithelium	+	Field, incidental, no disease reported (O.I.E., 2021)
African lion *Panthera leo* (n = 1)	–	+, bronchiole epithelium and bronchial gland	+	Field, respiratory disease (McAloose et al., 2020)
European lynx *Lynx lynx* (n = 1)	–	–	+	–
European badger *Meles meles* (n = 2)	–	+, bronchiole epithelium	n/a	–
Western gorilla *Gorilla sp*. (n = 1)	–	–	+	Field, cough (Gibbons, 2021)
Golden headed lion tamarin *Leontopithecus chrysomelas* (n = 1)	–	–	+	–
Common marmoset *Callithrix jaccus* (n = 1)	–	–	+	Experimental, only fever, no pneumonia or virus infection of lungs (Lu et al., 2020)

+Positive; ‐ negative; n/a, not available.

* Enterocytes unless indicated otherwise.

## 4 DISCUSSION

This study demonstrates the expression of ACE2 in the lungs of cattle, sheep, mink, European badgers, domestic cats, tigers and lions. The availability of ACE2 in the lungs of mink, cat, tigers and lions can be linked to the susceptibility of these species to develop pneumonia following SARS‐CoV‐2 infection (McAloose et al., 2020; Molenaar et al., 2020). Evaluation of ACE2 expression in the nasal mucosa of a limited number of species also reflected known differences in susceptibility to the virus. For example, productive infection of the nasal mucosal epithelium of the mink, ferret and cat with SARS‐CoV‐2 (Bosco‐Lauth et al., 2020; Everett et al., 2021; Molenaar et al., 2020) and the absence of nasal shedding in experimentally infected pigs (Vergara‐Alert et al., 2020). Importantly, understanding of the anatomical distribution of ACE2, particularly in the respiratory tract, clarified the disparities of host susceptibility previously investigated through binding of virus and with orthologous ACE2 proteins (Conceicao et al., 2020; Liu et al., 2021). The ubiquitous expression of ACE2 on the brush border of the small intestinal enterocytes in all investigated species demonstrates the conservation of ACE2 expression, which is similar to that found in humans (Ortiz et al., 2020).

Within the human lung, ACE2 is expressed on the luminal aspect of the cell membrane of the ciliated bronchiolar epithelial cells in the conducting airways (Hikmet et al., 2020; Lee et al., 2020b; Ortiz et al., 2020). Our study also demonstrated ACE2 immunolabelling in the ciliated bronchiolar epithelial cells of cattle, sheep, mink, badger, tigers and lions. Despite detection of low viral RNA copy numbers in nasal swabs of cattle following intranasal experimental inoculation of SARS‐CoV‐2 (Ulrich et al., 2020), other studies have successfully reported live virus replication in cattle lung explants (Di Teodoro et al., 2021). These differences could potentially be attributed to the large surface areas of the airways of cattle in relation to virus inoculum dose, or possibly lack of ACE2 expression in the first point of contact with virus inoculum, the nasal mucosa, similar to that observed in pigs.

The alveolus is the site of gaseous exchange in the lung and, as such, is highly important to the pathogenesis of SARS‐CoV‐2 infection. The epithelial lining of alveoli comprises two types of pneumocyte: terminally differentiated, flattened type I pneumocytes, which allow diffusion of gases, and plump type II pneumocytes, which produce surfactant and regenerate the epithelial lining following alveolar injury (Young et al., 2006). IHC and single‐cell RNA‐sequencing have shown that ACE2 is only expressed in approximately 1% of type II pneumocytes in humans (Ortiz et al., 2020). In the animal lungs examined, we only observed weak immunolabelling of type I pneumocytes of the cat, in contrast to a previous study which describes extensive expression of ACE2 within the respiratory tract of the cat and ferret (van den Brand et al., 2008). On the other hand, the hamster is widely used as a laboratory animal model to study the pathogenesis of SARS‐CoV‐2 due to high susceptibility to infection and its ability to reliably reproduce viral‐pneumonia (Chan et al., 2020; Imai et al., 2020). ACE2 could not be detected in the trachea and lung of hamsters in our study, as also reported by Suresh and colleagues (2021), despite using three different clones of commercial antibodies. Although immunolabelling of a positive kidney control tissue provided a good indication for cognate protein detection by IHC, the level of ACE2 expression could have been below the detection limits of the IHC techniques used. A previous study demonstrated low levels of ACE2 mRNA in hamster lungs by quantitative PCR (Suresh et al., 2021). In a highly susceptible species, such as the hamster, it is possible that the poor availability of ACE2 in the lung is compensated by the high binding affinity between SARS‐CoV‐2 spike proteins and host ACE2 to facilitate infection (Conceicao et al., 2020). Alternatively the binding and entry of SARS‐CoV‐2 in the hamster could be mediated by another cognate host receptor yet to be characterised.

In addition to ACE2 expression in the alveolar epithelium, ACE2 immunolabelling was observed in the endothelium and tunica media of the medium‐sized vessels within the alveoli of the dog and Sumatran tiger. In a separate study that evaluated IHC specificity of six commercially available anti‐ACE2 antibodies using human lung sections, including the antibody preparation used in our study, this commercial antibody produced robust staining in blood vessels including co‐localisation with CD31^+^ endothelial cells (Lee et al., 2020b). One of the COVID‐19 complications in human is thromboembolism (Bussani et al., 2020). Although there is immunohistologic evidence of alveolar blood vessels infected with SARS‐CoV‐2 and activation of the endothelial cells (expression of tissue factor, E‐selectin and VCAM‐1) in autopsied lungs (Bussani et al., 2020), an *in vitro* study has demonstrated only low susceptibility of cultured endothelial cells to SARS‐CoV‐2 infection (Hui et al., 2021). The upregulation of endothelial activation genes is also shown to be a consequence of pro‐inflammatory cytokines released from infected airway epithelial cells (Hui et al., 2021). Animals naturally infected with SARS‐CoV‐2, such as the mink (Molenaar et al., 2020) and cat (Carvallo et al., 2021), developed pulmonary injury evident by type I pneumocyte infection and damage. Currently, the significance of ACE2 expression in the vasculature of animals and the potential interaction with SARS‐CoV‐2 remains unclear.

The mucosal epithelium of the nasal cavity plays a critical role in SARS‐CoV‐2 transmission (Buitrago‐Garcia et al., 2020). A previous IHC study showed that 6 out of 12 human individuals expressed ACE2 in the epithelium of the nasal mucosa (Hikmet et al., 2020). In addition, ACE2 was abundantly distributed throughout the simple columnar (respiratory) epithelium of the human nasal mucosa, with the appearance of multifocal and discontinuous labelling on the cilia, but rare in the pseudo‐stratified columnar (olfactory) epithelium (Ortiz et al., 2020). In contrast, ACE2 is uniformly expressed on the respiratory epithelium within the nasal turbinate of the ferret and mink. The more widespread distribution of ACE2 in the nasal mucosa of these two Mustelinae could provide a larger area conducive for virus infection via respiratory droplet or aerosol, thus, facilitating virus transmission (Oude Munnink et al., 2021; Richard et al., 2020). In contrast, despite the sparse presence of ACE2 in the nasal mucosa of both the cat and hamster, the susceptibility of these species could be a consequence of the high binding affinity of SARS‐CoV‐2 to the cognate receptor (Conceicao et al., 2020). The nasal turbinate is often under‐ or not sampled in necropsies given the technical difficulties in extracting the tissues. Indeed, differences in the spatial expression of ACE2 in the upper (nasal turbinate) and lower (trachea and lung) respiratory tract underline the importance of examining these tissues to better understand potential binding sites within the respiratory tract.

In addition to the respiratory disease associated with COVID‐19, a substantial proportion of human patients also develop digestive symptoms and shedding of virus in faeces (Mao et al., 2020; Wu et al., 2020), which is supported by the ability of SARS‐CoV‐2 to infect enterocytes (Lamers et al., 2020; Zhou et al., 2020). Such dual tropism is also common among other coronaviruses of veterinary and zoonotic importance, such as bovine coronavirus, SARS‐CoV and MERS‐CoV (Leung et al., 2003; Saif & Jung, 2020; Zhou et al., 2017). SARS‐CoV‐2 has been detected in rectal swabs from dogs and cats living with COVID‐19 positive owners, although the viral load was much lower than in oropharyngeal swabs (Barrs et al., 2020; Sit et al., 2020). Similar findings have also been reported in several accounts of both natural and experimentally infected ferrets and mink (Everett et al., 2021; Gortázar et al., 2021; Oreshkova et al., 2020; Shuai et al., 2020). The diagnostic interpretation of a positive rectal swab in animals remains debatable as a positive result could suggest a productive infection in the gastrointestinal tract or ingestion of virus particles (Everett et al., 2021). Currently, no gastrointestinal disease has been reported from any of these animal species infected with SARS‐CoV‐2. However, considering that small intestinal enterocytes express high levels of ACE2 in these species, and given that allogrooming is an important social behaviour in many mammals (Carter & Leffer, 2015; Val‐Laillet et al., 2009), which likely facilitates oral ingestion of virus, virological surveillance from rectal swabs should be routinely undertaken in animals. One limitation of our study is that the large intestine was not examined. In humans, ACE2 is highly abundant in the small intestine but only low levels of ACE2 expression can be detected in the large intestine by IHC and transcriptomic analysis (Hikmet et al., 2020). ACE2 is reported to be absent in the large intestine of hamsters as determined by IHC (Suresh et al., 2021). Further investigation is needed to explore the spatial distribution of ACE2 in various regions of the gastrointestinal tract to determine other potential sites for ACE2‐mediated virus infection.

ACE2 of the domestic cat is highly effective in mediating SARS‐CoV and SARS‐CoV‐2 infection based on *in vitro* virus‐receptor binding studies (Conceicao et al., 2020; Liu et al., 2021). Although both SARS coronaviruses do not generally induce clinical disease in the cat, they can induce tracheobronchoadenitis evident at the microscopic level and this pathology can be related to ACE2 distribution in the respiratory tract (Gaudreault et al., 2020; van den Brand et al., 2008). In large felids, coughing has been reported in several Malayan tigers (*P. tigris jacksoni*), Siberian tigers and African lions infected with SARS‐CoV‐2, supported by radiographic evidence of peri‐bronchial consolidation and cytological detection of SARS‐CoV‐2‐infected tracheal epithelial cells (McAloose et al., 2020). In our study, we report the detection of ACE2‐positive bronchiolar epithelial cells in the lungs of Sumatran and Siberian tiger as well as African and Asiatic lions. The evidence of ACE2 expression in the conducting airway and clinicopathological reports of disease in large felids highlight the importance of appropriate biosecurity measures wherever these species are kept in captivity or handled during conservation research and management activities.

It is universally accepted that both mink and ferrets are susceptible to SARS‐CoV‐2 infection. Infection of both the upper and lower respiratory tract, supported by molecular, virological and pathological findings, has been observed in naturally and experimentally infected mink (Molenaar et al., 2020; Shuai et al., 2020). In contrast, only upper respiratory tract infection has been observed in ferrets experimentally infected with SARS‐CoV‐2 (Everett et al., 2021; Richard et al., 2020). Hence, the different spatial expression of ACE2 in the upper and lower respiratory tracts of the ferrets and mink is consistent with the observed difference in disease pathogenesis and transmission. Importantly, despite the poor binding of SARS‐CoV‐2 to ACE2 orthologues of the mink and ferret conducted *in vitro* (Conceicao et al., 2020; Liu et al., 2021), the unequivocal presence of ACE2 in the respiratory tract of both species promotes the permissibility for infection. These data also support the uniqueness of ferrets and mink to serve as suitable animal models in advancing our knowledge of both symptomatic and asymptomatic SARS‐CoV‐2 infection as well as therapeutic intervention evaluation (de Vries et al., 2021; Everett et al., 2021).

Most wild mustelids, including American and European mink (*Mustela lutreola*) and the wild cousin of the domestic ferret, the European polecat (*Mustela putorius*), live predominantly solitary lives, coming together only for breeding. Such circumstances would limit opportunities for viral transmission in the wild and are in stark contrast to the high‐density housing of mink on fur farms across Europe, North America and Asia. However, other species of mustelids including weasels, ferret badgers (*Melogale spp*.) and hog badgers (*Arctonyx collaris*) are consumed in parts of East Asia and may be found together in close proximity to other species in live animal markets (Dong et al., 2007; Woo et al., 2006; Xiao et al., 2021). Virological surveillance conducted at live animal markets following the 2003 SARS‐CoV outbreak identified the presence of other coronaviruses in the Chinese ferret‐badger (*Melogale moschata*), yellow‐bellied weasel (*Mustela kathiah*) and Siberian weasel (*Mustela sibirica*) (Dong et al., 2007). Evidence in the present study of ACE2 expression in the bronchiolar epithelial cells of two different subfamilies of Mustelidae (mink [Mustelinae] and European badger [Melinae]) suggests that analysis of the distribution of ACE2 in tissues and the binding affinity of SARS‐CoV‐2 in other mustelids may identify further susceptible species. More detailed examination of the distribution of ACE2 expression in the European badger may be particularly valuable as unlike other mustelids this species is highly social and can reach relatively high population densities in parts of their range (Johnson et al., 2002).

The emergence of SARS‐CoV‐2 is likely a consequence of virus evolution and positive selection of an ancestral bat coronavirus in an intermediate animal host which subsequently infected a human host (Andersen et al., 2020; Lau et al., 2005). For example, although SARS‐CoV has its origins in bats, the palm civet cat (*Paauma larvata*) was identified as the source of human infection during the 2003–2004 outbreak (Andersen et al., 2020; Lau et al., 2005). The intermediate host that may have led to the emergence and spread of SARS‐CoV‐2 in the human population is yet to be identified. It has been proposed that a likely candidate would be a species with a compatible and efficient ACE2 receptor that is kept at sufficiently high population density to facilitate virus selection, as is the case for farmed mink (Larsen et al., 2021; Zhou & Shi, 2021). Conventionally domestic animals, such as pigs, cattle and sheep, are often housed at high densities for intensive food production. However, the lack of ACE2 distribution in the respiratory tract of the pig, and the poor *in vivo* susceptibility of pigs, cattle and sheep (Ulrich et al., 2020; Vergara‐Alert et al., 2020), suggest that these species are unlikely to be effective intermediate hosts for coronavirus evolution.

A major concern is the potential for transmission of SARS‐CoV‐2 to free‐living wildlife and the subsequent establishment of a reservoir of infection providing the conditions for viral mutation, recombination and reintroduction to the human population (Hoffmann et al., 2021; Koopmans, 2021). Further studies are being undertaken, which should delineate the distribution of ACE2, specifically in the respiratory and gastrointestinal tract of other mammalian species. Additional information on the distribution of this viral receptor will enhance our knowledge of the susceptibility and likely importance of animals in the evolution and emergence of SARS coronaviruses.

## ETHICAL STATEMENT

No ethical approval was required as tissue blocks were derived from histology archives.

## AUTHOR CONTRIBUTION STATEMENT

F.Z.X.L., S.A., C.C., N.T. performed the experiments. F.Z.X.L., A.N., S.S., S.L.P., S.V., A.S.B., A.M.P.B. conducted formal analysis. F.Z.X.L., D.B., E.W., J.J., A.M.P.B., S.A. provided the methodology. A.N., R.J.D., W.H.M.P., I.H.B., A.R.F., S.M.B. provided with resources. F.Z.X.L. wrote the original draft. All authors reviewed and edited the manuscript.

## CONFLICT OF INTEREST

The authors declare no conflict of interest.

## Supporting information



Supporting information

Supporting information

Supporting information

Supporting information

## Data Availability

The data that support the findings of this study are available from the corresponding author upon reasonable request.
