# Supplementary figures and images for: Differential susceptibility of SARS‐CoV‐2 in animals: Evidence of ACE2 host receptor distribution in companion animals, livestock and wildlife by immunohistochemical characterisation
Source: Transbound Emerg Dis. 2021 Jul 26;69(4):2275–86. doi: 10.1111/tbed.14232 (PMC8447087; doi:10.1111/tbed.14232)

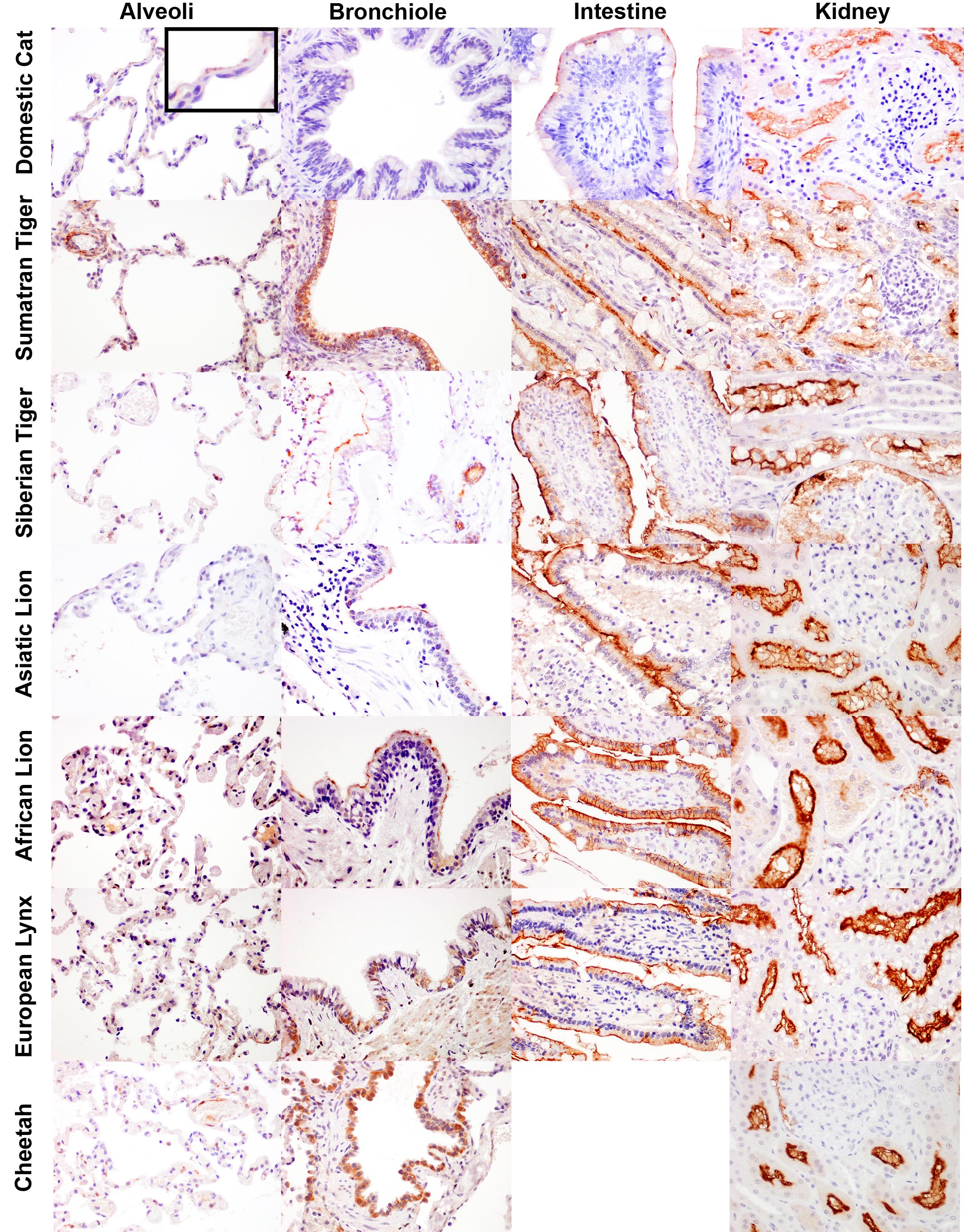

Supplement: Supplementary file 1 — Supporting information [file TBED-69-2275-s004.jpg]

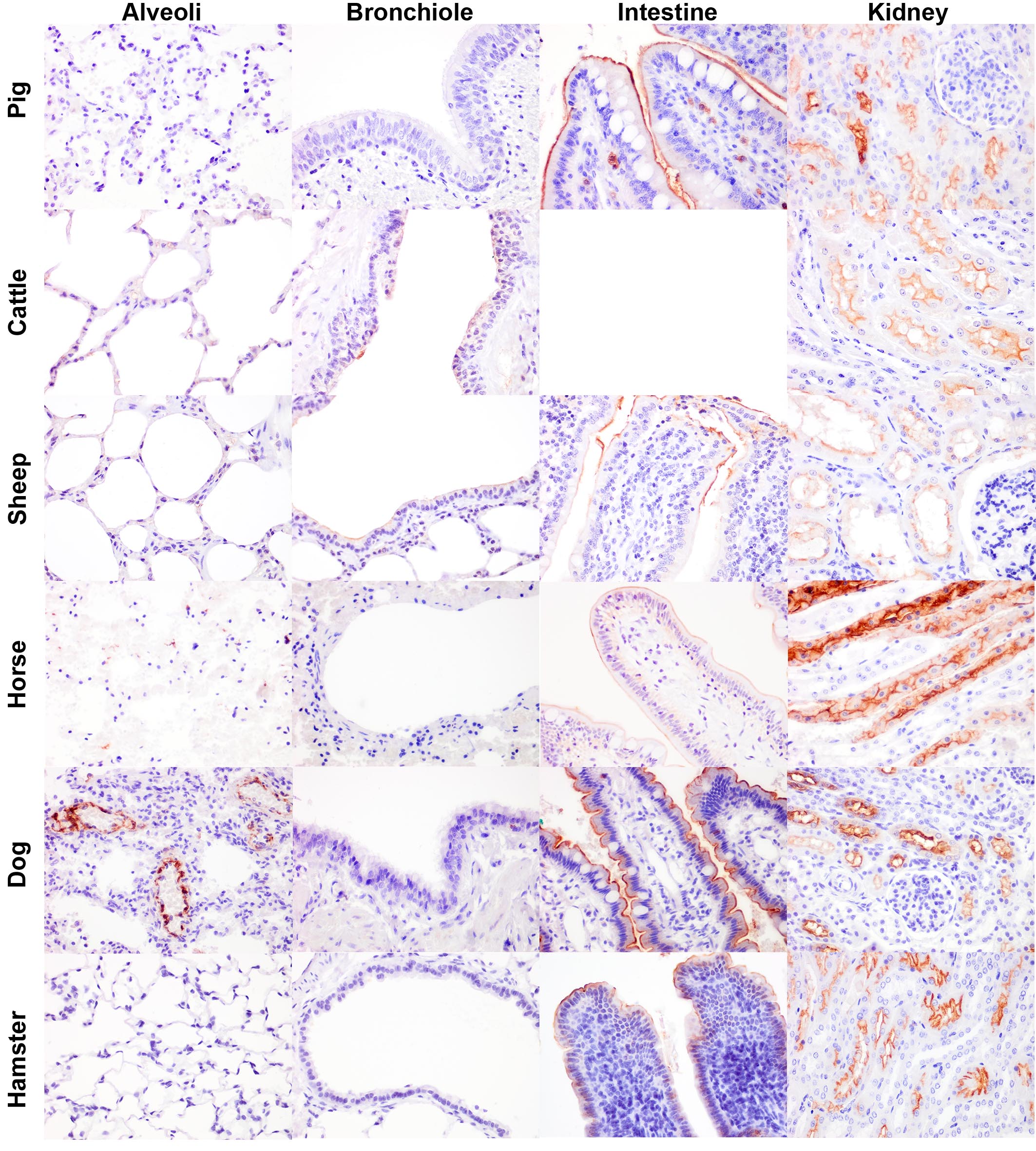

Supplement: Supplementary file 2 — Supporting information [file TBED-69-2275-s002.jpg]
