# Supplementary material for: Differential susceptibility of SARS‐CoV‐2 in animals: Evidence of ACE2 host receptor distribution in companion animals, livestock and wildlife by immunohistochemical characterisation
Source: Transbound Emerg Dis. 2021 Jul 26;69(4):2275–86. doi: 10.1111/tbed.14232 (PMC8447087; doi:10.1111/tbed.14232)
Supplement: Supplementary file 3 — Supporting information [file TBED-69-2275-s001.docx]

**S1 Table. Cross reactivity of human ACE2 polyclonal antibody with other mammalian ACE2 proteins on formalin-fixed paraffin-embedded sections**

| **Common name** | **Scientific Name** | **BHK-21 Transfected Cells** | **Kidney Sections** |
| --- | --- | --- | --- |
| Cat | *Felis catus* | + | + |
| Dog | *Canis lupus familiaris* | + | + |
| Ferret | *Mustela putorius furo* | + | + |
| Least horseshoe bat | *Rhinolophus pusillus* | + | N.D. |
| Little brown bat | *Myotis lucifugus* | + | N.D. |
| Pig | *Sus scrofa domestica* | + | + |
| Golden Syrian hamster | *Mesocricetus auratus* | + | + |
| African lion | *Panthera leo* | N.D. | + |
| American mink | *Neovison vison* | N.D. | + |
| Asiatic lion | *Panthera leo leo* | N.D. | + |
| Cheetah | *Acinonyx jubatus* | N.D. | + |
| Common marmoset | *Callithrix jaccus* | N.D. | + |
| Domestic cattle | *Bos taurus* | N.D. | + |
| European Badger | *Meles meles* | N.D. | + |
| European lynx | *Lynx lynx* | N.D. | + |
| Golden headed lion tamarin | *Leontopithecus chrysomelas* | N.D. | + |
| Horse | *Equus caballus* | N.D. | + |
| Sheep | *Ovis aries* | N.D. | + |
| Siberian tiger | *Panthera tigris altaica* | N.D. | + |
| Sumatran tiger | *Panthera tigris sondaica* | N.D. | + |
| Western gorilla | *Gorilla sp.* | N.D. | + |

N.D. = not determined
