# Supplementary material for: Differential susceptibility of SARS‐CoV‐2 in animals: Evidence of ACE2 host receptor distribution in companion animals, livestock and wildlife by immunohistochemical characterisation
Source: Transbound Emerg Dis. 2021 Jul 26;69(4):2275–86. doi: 10.1111/tbed.14232 (PMC8447087; doi:10.1111/tbed.14232)
Supplement: Supplementary file 4 — Supporting information [file TBED-69-2275-s003.docx]

**S2 Table. Amino acid homology alignment against ACE2 immunogen sequence used for raising the rabbit polyclonal antibody**

| **Common name** | **Scientific Name** | **Peptide Sequence Identification** | **Amino Acid Sequence (Positions 788 to 805)** | **Identity (%)** |
| --- | --- | --- | --- | --- |
| Human | *Homo sapiens* | Synthetic Peptide, Abcam | KGENNPGFQNTDDVQTSF | n/a |
| Alpaca | *Vicugna pacos* | XP_006212709.1 | KGENNSGFQNGDDVQTSF | 89 |
| American Mink | *Neovison vison* | QPL12211.1 | KGENNPGFQNVDDVQTSF | 94 |
| Cattle | *Bos taurus* | NP_001019673.2 | KGENNSGFQNIDDVQTSL | 83 |
| Cheetah | *Acinonyx jubatus* | XP_026910300.1 | KGENNPGFQHADDVQTSF | 89 |
| Common marmoset | *Callithrix jaccus* | XP_008987241.1 | KGEDNPGFQNSEEVQTSF | 78 |
| Cat | *Felis catus* | XP_023104564.1 | KGENNPGFQHADDVQTSF | 89 |
| Dog | *Canis lupus familiaris* | NP_001158732.1 | KGENNPGFQSGDDVQTSF | 89 |
| Ferret | *Mustela putorius furo* | NP_001297119.1 | KGENNPGFQNVDDVQTSF | 94 |
| Horse | *Equus caballus* | XP_001490241.1 | KGENNPGFQNGDDVQTSF | 94 |
| Least horseshoe bat | *Rhinolophus pusillus* | ADN93477.1 | KGENNPGFQNGDDVQTSF | 94 |
| Leopard | *Panthera pardus* | XP_019273509.1 | KGENNPGFQHADDVQTSF | 89 |
| Little brown bat | *Myotis lucifugus* | XP_023609438.1 | KGENNPGFQNGDDVQTSF | 94 |
| Pig | *Sus scrofa domestica* | XP_020935033.1 | KGESNSGFQNGDDIQTSF | 78 |
| Sheep | *Ovis aries* | XP_011961657.1 | KGENNSGFQNTDDVQTSL | 89 |
| Siberian tiger | *Panthera tigris altaica* | XP_007090142.1 | KGENNPGFQHADDVQTSF | 89 |
| Golden Syrian hamster | *Mesocricetus auratus* | XP_005074266.1 | KGESNAGFLSNDDAQTSF | 67 |
| Western gorilla | *Gorilla gorilla* | XP_018874749.1 | KGENNPGFQNTDDVQTSF | 100 |

n/a = not applicable
